# Potential of a Quorum Quenching Bacteria Isolate *Ochrobactrum intermedium* D-2 Against Soft Rot Pathogen *Pectobacterium carotovorum* subsp. *carotovorum*

**DOI:** 10.3389/fmicb.2020.00898

**Published:** 2020-05-08

**Authors:** Xinghui Fan, Tian Ye, Qiting Li, Pankaj Bhatt, Lianhui Zhang, Shaohua Chen

**Affiliations:** ^1^State Key Laboratory for Conservation and Utilization of Subtropical Agro-bioresources, Guangdong Province Key Laboratory of Microbial Signals and Disease Control, Integrative Microbiology Research Centre, South China Agricultural University, Guangzhou, China; ^2^Guangdong Laboratory for Lingnan Modern Agriculture, Guangzhou, China

**Keywords:** quorum sensing, quorum quenching, degradation, *N*-acyl homoserine lactone, *Ochrobactrum intermedium*

## Abstract

Quorum quenching (QQ) is a promising strategy for preventing and controlling quorum sensing (QS)-mediated bacterial infections. It interferes with QS by the inhibition of signal synthesis, the detection of enzyme-catalyzed degradation, and the modification of signals. *N*-Acyl homoserine lactones (AHLs) represent a family of widely conserved QS signals involved in the regulation of virulence factor production in many Gram-negative bacterial pathogens. In this study, AHL-degrading bacterial strains were isolated, and the most efficient one was evaluated for its potential against QS-mediated pathogens. Results showed that an AHL-degrading bacteria *Ochrobactrum intermedium* D-2 effectively attenuated maceration produced by the pathogen *Pectobacterium carotovorum* subsp. *carotovorum* (Pcc) on radish and potato slices. Strain D-2 exhibited a superior AHL degradation activity and efficiently degraded various AHLs, including *N*-hexanoyl-L-homoserine lactone (C6HSL), *N*-(3-oxohexanoyl)-L-homoserine lactone (3OC6HSL), *N*-(3-oxooctanoyl)-L-homoserine lactone (3OC8HSL), and *N*-(3-oxododecanoyl)-L-homoserine lactone (3OC12HSL). Analysis of the degradation products of AHL by gas chromatography-mass spectrometry led to the identification of *N*-cyclohexyl-propanamide and propanamide as the main intermediate products, suggesting that AHL was degraded by hydrolysis. Annotation and analysis of the whole genome sequence of strain D-2 revealed the presence of an AHL-lactonase, termed AidF. Moreover, the application of strain D-2 was able to substantially reduce the disease severity caused by Pcc on host plants. These results reveal the biochemical basis of a highly efficient AHL-degrading bacterial isolate and present the potential to attenuate Pcc virulence through QQ.

## Introduction

The gram-negative strain *Pectobacterium carotovorum* subsp. *carotovorum* (Pcc) (formerly known as *Erwinia carotovora* subsp. *carotovora*) is a broad host range pathogen that causes soft rot, wilt, and blackleg in various cruciferous vegetables such as Chinese cabbage, potato, carrot, tomato onion, rhubarb, and cucumber (Lim et al., 2013). Recently, Pcc has been reported to cause wet rot disease in banana plants (Basim et al., 2019). In 2016, severe wet rot (tip over) in banana plants caused by Pcc was recorded in greenhouses in Turkey (Basim et al., 2019). Pcc is also a major pathogen of tuber soft rot in different countries and results in severe economic losses (Muturi et al., 2018; Naas et al., 2018).

At high cell densities, bacteria communicate with each other through a quorum sensing (QS) system to perform certain collective functions (Deng et al., 2013; Kalia et al., 2019). A wide range of microbes uses the QS system as a defense or attack mechanism for antibiotics production, bioluminescence expression, biofilm formation, or the expression of virulence and pathogenicity factors (Deng et al., 2011; Liu et al., 2015; An et al., 2020). There are several distinguished QS systems: the *N*-acyl homoserine lactone (AHL) QS system, the diffusible signal factor (DSF) QS system, the autoinducing peptide (AIP) QS system, and the autoinducer-2 (AI-2) QS system (Waters and Bassler, 2005; Zhou et al., 2017). The attack mechanism and expression of virulence and pathogenicity initiate after sensing and binding the signal molecules to receptors (Jayaraman and Wood, 2008; Deng et al., 2011). Therefore, the interruption of the “attack mechanism” in QS-mediated pathogens is a feasible strategy to protect hosts against infectious diseases.

The disruption of QS is termed quorum quenching (QQ), which has been suggested as a potential strategy for disease control (Chen F. et al., 2013). Theoretically, QQ can be achieved by inhibiting the synthesis or detection of signal molecules and by the enzyme-catalyzed degradation or modification of signal molecules (LaSarre and Federle, 2013; Grandclement et al., 2016). The application of QQ bacteria and the enzyme-based degradation of the signal molecule are two of the most extensively studied non-toxic methods of QQ. Identified QQ bacteria and enzymes target AHL, DSF, AI-2, and 2-heptyl-3-hydroxy-4-quinolone (Pustelny et al., 2009; Roy et al., 2010; Pereira et al., 2013; See-Too et al., 2017; Ye et al., 2019, 2020; Wang et al., 2020). Strains inactivating the QS signal are known as QQ strains that can produce and secrete QQ enzymes (lactonases, acylases and oxidoreductase) to inactivate signaling molecules (Uroz et al., 2009; Fetzner, 2015; See-Too et al., 2017). QQ strains are agriculturally essential to attenuate the virulence of pathogenic bacteria (Grandclement et al., 2016).

AHLs represent a family of widely conserved QS signals involved in the regulation of virulence factor production in many Gram-negative bacterial pathogens including Pcc (Whitehead et al., 2001). Several QQ strains and their AHL-degrading enzymes have been reported to attenuate the virulence of Pcc. *Mesorhizobium* sp. and *Lysinibacillus* sp. are known to significantly reduce Pcc pathogenicity and suppress tissue maceration (Mahmoudi et al., 2011; Garge and Nerurkar, 2016). Recently, AHL-degrading QQ bacteria have been isolated and characterized as *Sphingomonas*, *Diaphorobacter*, *Sphingopyxis*, *Acidovorax, Stenotrophomonas*, *Bosea*, and *Mycobacterium* (Molina et al., 2003; Carlos Reina et al., 2019; Kusada et al., 2019; Zhang et al., 2019). Previous studies have suggested that QQ strains can be applied as biocontrol agents to protect hosts against bacterial diseases in agriculture (Chen et al., 2011a, b; Garge and Nerurkar, 2016).

Extensive tissue maceration is a specific symptom of soft rot disease by Pcc (Pirhonen et al., 1993). Pcc pathogenicity mainly depends upon the abundance of plant cell wall-degrading exoenzymes, including pectate lyase (Pel), pectin lyase (Pnl), polygalacturonase (Peh), cellulase (Cel), and protease (Prt) (Joshi et al., 2016). The production of exoenzymes in Pcc is modulated by a cell-density-dependent system known as QS through a signal molecule 3OC6HSL. It has been reported that 3OC6HSL concentration increases with the Pcc population that triggers the expression of exoenzyme-synthesizing genes (Joshi et al., 2016). Two signaling chemicals (AHL and AI-2) of *Pectobacterium carotovorum* play a role in QS (Pollumaa et al., 2012). During pathogenesis, QS is regulated by transcriptional and post-transcriptional regulatory factors (Fuqua et al., 1994).

The aim of the present study was to isolate and characterize new AHL-degrading bacteria to develop QQ strains. A new QQ strain D-2 was identified along with the degradation pathway. Furthermore, the properties of strain D-2 was investigated to establish control measures against AHL-dependent bacterial pathogens. This study suggests a new area of research for the development of plant disease control.

## Materials and Methods

### Bacterial Strains, Chemicals, and Media

Pure C6HSL, 3OC6HSL, and 3OC12HSL were obtained from Shanghai UDChem Technology Co., Ltd. 3OC8HSL was purchased from Sigma Aldrich Chemicals Co., Ltd (Shanghai, China). The chemical structures of various *N*-acyl homoserine lactones (AHLs) used in this study are shown in Supplementary Figure S1. Different antibiotics—ampicillin (Amp, 50 mg⋅mL^–1^), chloramphenicol (Cm, 30 mg⋅mL^–1^), tetracycline (Tc, 5 mg⋅mL^–1^), streptomycin (Str, 50 mg⋅mL^–1^), gentamicin (Gen, 50 mg⋅mL^–1^), and kanamycin (Kan, 50 mg⋅mL^–1^)—were used in antimicrobial susceptibility tests. Pesticide streptomycin (98% active ingredient) was used as a positive control at a concentration of 3.3 g⋅mL^–1^. Bacterial strains and plasmids used in this study are presented in Table 1.

**TABLE 1:** Bacterial strains and plasmids used in this study.

Strains or Plasmids	Characteristics	Source
**Bacteria strains**		
*Pectobacterium carotovorum* subsp. *carotovorum* Z3-3	Wild type	(See-Too et al., 2017)
*Escherichia coli*		This lab
*Escherichia coli* DH5*α*	Standard cloning host, host of pET28b-*aidf*	This lab
*Escherichia coli* BL21 (DE3)	The expression host, host of pET28b-*aidf*	This lab
*Ochrobactrum intermedium* D-2	Wild type, AHL-degrading strain	This lab
*Bacillus thuringiensis* subsp. *israelensis* B23	Control strains	This lab
*Agrobacterium tumefaciens* NT1	Biosensor	(Farrand et al., 2002; Steindler and Venturi, 2007)
**Plasmids**		
pET28(b)	Expression vector (T7 promoter, His/Thrombin/T7 tag, Kan ^r^)	This lab
pET28b-*aidf*	pET28 (b) derivative carrying *aidf* gene	This study

The bacterial strain was cultured in minimal salt medium (MSM) [composition (g⋅L^–1^): (NH_4_)_2_SO_4_ 2.0, Na_2_HPO_4_⋅12H_2_O 1.5, KH_2_PO_4_ 1.5, MgSO_4_⋅7H_2_O 0.2, CaCl_2_⋅2H_2_O 0.01, FeSO_4_⋅7H_2_O 0.001, pH 6.5] and Luria-Bertani medium (LB) [composition (g⋅L^–1^): Yeast extract 5.0, Tryptone 10.0, NaCl 10.0, pH 7.0] (Chen et al., 2011a). Minimal medium (MM) [composition (g⋅L^–1^): (NH_4_)_2_SO_4_ 2.0, MgSO_4_⋅7H_2_O 0.2, CaCl_2_ 0.01, FeSO_4_ 0.005, MnCl_2_ 0.002, K_2_HPO_4_ 10.5, KH_2_PO_4_ 4.5, mannitol 2, glycerol 2, pH 6.5] was used for detecting AHL (Liu et al., 2017). Agar (15 g⋅L^–1^) was used to solidify the medium. Media were sterilized at 121 °C for 20 min.

### Screening and Identification of Quorum Quenching Bacterial Isolates

Approximately 30 g soil samples were collected from a power plant (latitude 36°50′; longitude 113°52′) in Power Plant Road, Xingtai City, Hebei Province, China, and the samples were stored at 4°C before experiments.

QQ strains were isolated from soil samples after the enrichment culture with 3OC6HSL as the sole carbon and nitrogen source. The isolation and purification procedure was carried out as follows (Ye et al., 2019). Five-gram samples were added to 20 mL of MSM medium containing 5 μM 3OC6HSL and cultivated at 30°C for 7 days with shaking condition at 200 rpm. After 7 days, 2 mL of suspension was transferred to another 20 mL of fresh MSM medium containing 10 μM 3OC6HSL and cultivated under the same conditions for 7 days. The procedure was repeated until the 3OC6HSL concentration increased to 200 μM. The final suspension was diluted to different concentrations (10^–1^–10^–7^). These dilutions were then placed in LB medium plates and incubated at 30°C for 24∼48 h. Colonies with different characteristics were purified with the agar streak method (Huang et al., 2020).

The ability of isolates to inactivate 3OC6HSL was evaluated by detecting AHL through a biosensor, *Agrobacterium tumefaciens* NT1 (Piper et al., 1993; Zhang et al., 2019). Briefly, MM medium plates containing 5-bromo-4-chloro-3-indolyl-β-D-galactopyranoside (X-Gal; 40 μg⋅mL^–1^) were used for the evaluation of QQ activity. The MM medium plates were cut into separate slices (1 cm in width). The LB cultures of different isolates were mixed with 3OC6HSL (40 μM⋅mL^–1^). These mixtures were cultivated at 28 °C for 1 h, and then streaked to one end of the agar slices, respectively. After that, the new cultures (OD_600_ = 0.1) of the AHL biosensor strain, *A. tumefaciens* NT1, were spotted at progressively further distances from these mixtures. The plates were incubated at 28°C for 24 h in the dark. The gene modulated the synthesis, and the secretion of *β*-galactosidase began to express when the biosensor strain detected 3OC6HSL possessing diffusivity and diffusing from the end of the agar slices. X-Gal supplemented in MM strips was transformed into galactose and a dark blue substance, which makes biosensor colonies blue through enzymolysis. The diffusion length of 3OC6HSL and the blue colonies increases with the increase of 3OC6HSL concentration. Therefore, the length of the blue spots was applied to determine which isolates are more effective.

The ability of isolates to degrade 3OC6HSL was further determined by high-performance liquid chromatography (HPLC). To screen, the isolates were grown in MSM containing 1 mmol⋅L^–1^ 3OC6HSL as the sole source of carbon for 24 h at 30°C and 200 rpm. After 24 h, the remaining amount of 3OC6HSL was extracted and analyzed by HPLC. The experiment was performed in triplicate with non-inoculated samples as control.

QQ strains were characterized through analysis of morphological, physiological, biochemical, and molecular characterization. Morphological features of QQ strains were observed on LB medium plates after 24 h incubation at 30°C. Colony and cellular morphologies of QQ strains were investigated under an electron microscope (BH-2 Olympus, Japan) and scanning electron microscope (XL-30 ESEM, Philips Optoelectronics Co., Ltd., Holland) on LB plates and slides, respectively. Molecular characterization was carried out by targeting the 16S rDNA gene with universal primers, 27F (5′-AGAGTTTGATCCTGGCTCAG-3′) and 1492R (5′-GGTTACCTTGTTACGACTT-3′) (Chen et al., 2011b; Zhang et al., 2019). Purified PCR product was sequenced from Shanghai Invitrogen Technology Co. Ltd., China. Sequence similarity with reported bacterial strains was assessed through the BLAST tool of NCBI (https://blast.ncbi.nlm.nih.gov/Blast.cgi). Multiple nucleotide sequence alignment and phylogenetic analysis were performed with the approach of the neighbor-joining algorithm in MEGA X 10.0.2 (Silva et al., 2005; Evans et al., 2006; Chen S. et al., 2013; Yang et al., 2018).

The antimicrobial susceptibility test of strain D-2 was performed for further molecular assays. D-2 was inoculated into 1 mL of LB medium and incubated overnight at 30°C and 200 rpm for 12–16 h. Cultures were added to LB medium containing various antibiotics at different concentrations and incubated at 30°C and 200 rpm for 12 h. Antibiotics such as ampicillin (Amp), chloramphenicol (Cm), tetracycline (Tc), streptomycin (Str), gentamicin (Gen), and kanamycin (Kan) were used in this experiment at concentrations of 5, 10, 20, 30, 40, 50, 100, 150, 200, 250, 300, 350, and 400 μg⋅mL^–1^. Three replicates were prepared for each treatment. The growth of Strain D-2 against antibiotics at different concentrations was measured by absorbance at 600 nm.

### Antagonistic Assay

Antagonistic interactions between isolate D-2 and pathogen Z3-3 were studied on the LB plate according to the method of Garge and Nerurkar (2017) with some modifications. Cultures of Strain D-2 were obtained after 12 h cultivation at 30°C and 200 rpm in LB medium. Cultures were extracted three times with ethyl acetate, and the organic phase was evaporated to dryness. Metabolites of strain D-2 were dissolved in 2 mL of methyl alcohol. LB agar plates containing pathogen Z3-3 were punched with a sterilized perforator, and 20 μL of the metabolite solution were injected into the holes. Twenty microliters of methyl alcohol were injected into the holes on another LB agar plate as control. Samples were observed for inhibition zones in the plate after incubation at 30°C for 24 h. Treatments were prepared in triplicate, and samples injected with methyl alcohol served as controls.

### Substrate Spectrum and Efficiency of QQ Strain D-2

Three types of synthetic AHLs (3OC6HSL, 3OC8HSL, and 3OC12HSL) were used in inactivation assays to investigate the substrate spectrum of QQ strain D-2. Strain D-2 was cultured overnight in LB, and its cells were harvested by centrifugation. Cell pellets were washed twice with phosphate buffer saline (PBS) and resuspended in MSM (pH 6.5) supplemented with different synthetic AHLs at a final concentration of 20 μmol⋅L^–1^. After the incubation at 30°C and 200 rpm for 24 h, a 5 μL mixture was placed on top of MM strips containing 40 μg⋅mL^–1^ X-gal followed by the bacterial biosensor. *B. thuringiensis* subsp. *israelensis* B23 and *E. coli* DH5*α* served as positive and negative controls, respectively.

Cells (obtained as mentioned above) were inoculated into 20 mL of MSM containing 3OC6HSL at a concentration of 1 mmol⋅L^–1^ and cultivated at 30°C and 200 rpm. Samples were collected at the same interval, and strain growth was measured at OD_600_. 3OC6HSL residues were extracted thrice with ethyl acetate, dried, and resuspended in 2 mL of acetonitrile for HPLC analysis. Three replications were prepared for each treatment, and samples without the QQ strain served as control. Control samples were also used to determine the abiotic loss of 3OC6HSL. HPLC was used for the quantification of 3OC6HSL. To detect 3OC6HSL residues, a 20 μL sample was injected into the Water e2690 HPLC system equipped with a Phenomenex C_18_ reversed-phase column (250 μm × 4.6 mm × 5 μm) and an array detection at 210 nm. The mixture of acetonitrile and water (30:70, *v/v*) was applied as a mobile phase at a flow rate of 0.5 mL⋅min^–1^ (Zhan et al., 2018).

### Maceration Attenuating Assay

Maceration attenuating experiments were performed to evaluate the suppressive and preventive effect of the QQ strain D-2 against Pcc by following the method of Ye et al. (2019). Healthy radish and potato tubers were obtained from the local market. Radish and potato tubers were washed three times with tap water and surface sterilized by sequentially immersing in 44% (*v/v*) sodium hypochlorite and 75% (*v/v*) ethanol for 60 s. Finally, tubers were rinsed with sterile distilled water and cut into slices of 0.5 cm. QQ strain D-2, *E.coli* DH5*α*, B23, and Pcc Z3-3 were cultured overnight in LB medium, and cells were harvested by centrifugation. Cell pellets were washed twice and resuspended in PBS buffer (pH 7.0).

To conduct the assay, QQ strain D-2 (1.86 × 10^11^ CFU⋅ml^–1^), *E.coli* DH5*α* (1.86 × 10^12^ CFU⋅ml^–1^), B23 (1.86 × 10^11^ CFU⋅ml^–1^), and agricultural streptomycin (3.3 g⋅ml^–1^) were separately mixed with Pcc Z3-3 (1.2 × 10^5^ CFU⋅ml^–1^) at the same volume, and 2 μL of each mixture was injected into tuber slices. *B. thuringiensis* subsp. *israelensis* B23 and *E. coli* DH5*α* served as positive and negative controls, respectively. Slices of all treatments were placed in pallets and cultivated at 28°C for 24 h in an artificial climate box. All treatments consisted of three replicates, and experiments were repeated five times. In order to express disease severity numerically, the diameter of the macerated region (square millimeters) was measured to calculate the percentage in comparison to pre-inoculation tissue.

### Identification of Biodegradation Products of AHL

QQ strain D-2 was cultured overnight in LB medium (OD_600_ = 1.0∼1.5), cells were harvested by centrifugation at 10,000 rpm for 1 min, and the supernatant was discarded. Cell pellets were washed twice with PBS buffer and resuspended in MSM (pH 6.5) containing 1 mmol⋅L^–1^ AHL, and cultivated at 30°C and 200 rpm for 32 h. Samples were collected at 0, 8, 16, and 32 h. AHL was extracted from the samples three times with ethyl acetate, followed by drying and resuspension in 2 mL of methanol for HPLC analysis. Three replicates were prepared for each treatment.

To determine the AHL degradation metabolism, gas chromatography-mass spectrometry was carried out in an Agilent (Agilent 6890N/5975) HP-5MS capillary column (30 m × 250 μm × 0.25 μm). A flow rate of carrier gas (helium) was kept at 1 mL⋅min^–1^. Temperatures of the injector, the ion source, the GC-MS interface, and quadrupole were set as 200, 230, 280, and 150 °C, respectively. The initial temperature of the column was held at 150°C for 2 min, raised to 280°C at the rate of 25°C per minute, and held for 3 min. Metabolites were analyzed at a split ratio of 5:1 and an injection volume of 1 μL. Ionization was conducted in the electron impact (EI) mode at 70 eV. Metabolites were identified by comparing at the National Institute of Standards and Technology (NIST, United States) library database (Chen et al., 2014).

### Whole-Genome Sequencing and Annotation

The whole genomic DNA of strain D-2 was extracted using EasyPure^®^ Bacteria Genomic DNA Kit (TransGen Biotech, Beijing, China), and the concentration and purity of DNA were detected using a NanoDrop-2000 spectrophotometer. The sequencing of the complete genome was executed by LC-BIO, Technology Co. Ltd (Hangzhou, China) using a hybrid of the HiSeq 2500 (Illumina, United States) and PacBio RS II (Pacific Biosciences, United States) platforms. The coding genes were annotated with the National Center for Biotechnology Information (NCBI) NR database by BLAST. The functions of coding genes were then annotated by the Gene Ontology (GO) database, and the pathways were annotated using the Kyoto Encyclopedia of Genes and Genomes (KEGG) database. The proteins encoded by genes were classified and annotated on databases of Clusters of Orthologous Groups (COG/KOG) and Carbohydrate-Active enzyme (CAZy).

### Determination, Expression, and Purification of AidF Protein

The whole genomic DNA of strain D-2 was sequenced, and the prediction of putative coding sequences and gene annotations were performed. To determine the potential QQ enzyme of strain D-2, the homology comparison between the AidH enzyme, a known QQ enzyme from *Ochrobactrum* sp. T63 (Mei et al., 2010) and putative proteins of strain D-2 was performed through the BLAST tool of NCBI.

The candidate QQ gene, *aidf*, of *Ochrobactrum intermedium* D-2 was amplified by PCR using the designed primer pair forward (5′- CAGCAAATGGGTCGGGAT CCGATGACGATCAATTATCGCGA-3′) and reverse (5′- TGGTGGTGGTGGTGCTCGAGTCAAACGGTGCAGTCGCG -3′) (BamH I and Xho I sites are underlined). PCR was performed using the following cycling parameters: 98°C for 10 s, 63°C for 10 s, and 72°C for 1 min, for 35 cycles. After electrophoretic separation, amplified DNA fragments were purified using MN NucleoSpin Gel and PCR Clean-up. The purified PCR products were cloned into the expression vector (pET-28b) digested with BamH I and Xho I and transformed into *E. coli* DH5*α*. The constructed plasmids (pET-28b-*aidf*) were extracted from the strain *E. coli* DH5*α* pET-28b- *aidf* and transformed into *E. coli* BL21(DE3) (Novagen).

The recombinant strain *Escherichia coli* BL21(DE3) pET-28b-*aidf* was cultured in 5 mL of fresh LB medium. When the OD_600_ of the transformants reached 0.6, IPTG (final concentration, 0.8 mM) was added to induce protein expression. After induction for 12 h at 18°C, the mixture of its culture and 3OC6HSL (final concentration, 40 μM) was incubated at 30°C for 1 h. The inactivation activity was evaluated by the biosensor, as described above.

Furthermore, *E. coli* BL21 (DE3) pET-28b-*aidf* was used for the expression and purification of His-tagged AHL lactonase (AidF). For the expression and purification of His-tagged AidF, the full-grown culture of *E. coli* BL21 (DE3) harboring expressing plasmid was inoculated into 600 mL of LB medium containing kanamycin (50 mg⋅mL^–1^), and 0.8 mM IPTG (isopropyl-*β*-D-thiogalactoside) was added to LB medium when cultures had grown to an optical density at 600 nm (OD_600_) of 0.6 at 37°C, 200 rpm. After cultivation for 16 h at 18°C, the cells were harvested by centrifugation and resuspended with a binding buffer (20 mM sodium phosphate buffer, 500 mM NaCl, and 20 mM imidazole, pH 7.4). The proteins were released from the cells by sonication, and the supernate was employed in protein purification after centrifugation at 4 °C.

Protein purification was performed with the ÄKTA start systems (GE Healthcare, Tokyo, Japan). The protein sample was loaded on a HisTrap affinity chromatography column (GE Healthcare) equilibrated with a binding buffer and eluted with the 20% eluting buffer (20 mM sodium phosphate buffer, 500 mM NaCl, 500 mM imidazole, pH 7.4) after washing off unbound bacterial proteins. The expression and purification of the His-tagged AidF were assessed by SDS-PAGE analysis.

### Re-Lactonization Assay

To confirm whether the inactivation/utilization of 3OC6HSL by QQ strain D-2 and AidF protein is due to the opening lactone rings, 0.2 M HCL was added to the mixture of cells, along with 3OC6HSL in MSM after 24 h of incubation or a mixture of AidF protein and 3OC6HSL in a PBS buffer after 1 h of incubation. Acidification was carried out to enhance the re-lactonization of opened lactone rings (Yates et al., 2002). The 3OC6HSL of the acidified mixture was detected using a biosensor, as mentioned above.

### Statistical Analysis

Experimental data were analyzed by one-way analysis of variance (ANOVA), and means were compared by Bonferroni’s multiple comparison test in Graphpad Prism (Version 6.0). Experiments were arranged as a completely randomized design, and *P*-values < 0.05 were considered statistically significant.

## Results

### Isolation and Identification of QQ Strain D-2

According to the results of soil enrichment culture, eight morphologically different strains (grown on MSM containing 20 μmol⋅L^–1^ AHL) were attained by the streaking plate method and named as D-1∼8, respectively. These strains could inactivate/utilize AHL, and D-2 was the most effective among eight strains (Supplementary Table S1). Strain D-2 completely degraded AHL (1 mmol⋅L^–1^) within 24 h. Thus, strain D-2 was selected for further studies.

**FIGURE 1:**
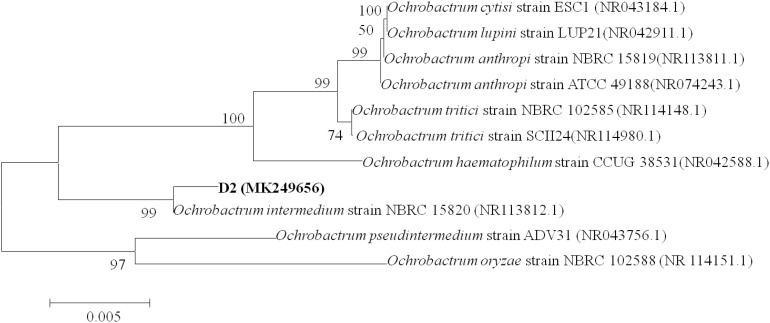
Phylogenetic tree based on 16S rDNA sequences of strain D-2 and representative *Ochrobactrum* species. Numbers in parentheses represent Genbank accession number. Numbers at the nodes indicate bootstrap values. Bar represents sequence divergence.

Colonies of strain D-2 appeared as white, small, round, convex, and smooth with neat edges when grown on an LB plate (Supplementary Figures S2A,B). SEM revealed cells as rod-shaped and motile with polar flagella. Dimensions of the cells were noted as 0.8–1.1 μm in length and 0.2–0.3 μm in width (Supplementary Figures S2C,D). The 16S rDNA gene of strain D-2 contains 1396 bp, and its accession number is MK249656. Analysis of the 16S rDNA gene sequence revealed that strain D-2 belongs to *Ochrobactrum*. Furthermore, isolate D-2 has high similarity (≥98%) with *Ochrobactrum intermedium* (accession number: NBRC 15820) (Figure 1). Further biochemical tests indicated that strain D-2 is Gram-negative and strictly aerobic. The strain was positive for oxidase and catalase, but negative for the anaerobic test and gelatin liquefaction (Supplementary Table S2). In the Biolog test, strain D-2 positively assimilated sodium butyrate, D-maltose, D-trehalose, gentiobiose, sucrose, D-turanose, *N-*acetyl-D-glucosamine, and *α*-D-Glucose. However, strain D-2 could not utilize dextrin, stachyose, D-raffinose, *α*-D-lactose, D-melibiose, β-methyl-D-glucoside, 3-methyl glucose, and D-fructose-6-PO4 (Supplementary Table S2). Therefore, strain D-2 was confirmed as *Ochrobactrum intermedium* based on the morphology, 16S rDNA gene analysis, physio-biochemical properties, and the Biolog microbial identification system. The strain was deposited in the Guangdong Microbial Culture Collection Center (GDMCC) (collection number: GDMCC 60409).

An antimicrobial susceptibility test of strain D-2 was carried out for further molecular assays. Supplementary Figure S3 indicates the sensitivity of strain D-2 against different antibiotics. The resistance of strain D-2 to ampicillin (Amp) was noted up to a concentration of 400 μg⋅mL^–1^. Strain D-2 exhibited resistance against kanamycin (Kan) up to a concentration of 350 μg⋅mL^–1^, at 40 μg⋅mL^–1^ against gentamicin (Gen) and streptomycin (Str), and at 10 μg⋅mL^–1^ against chloromycetin (Cm). Strain D-2 was not found to be resistant to tetracycline (Tc).

### QQ Strain D-2 Did Not Show Antagonism Against Pcc

To determine the antagonistic interactions between strain D-2 and pathogen Z3-3, metabolites of strain D-2 was injected into the hole on an LB agar plate containing pathogen Z3-3. Meanwhile, methyl alcohol was injected into another hole on the same LB agar plate supplemented with pathogen Z3-3 as control. The results of the antagonism assay indicated that both groups inoculated with methyl alcohol (Supplementary Figure S4A) and strain D-2 metabolites (Supplementary Figure S4B) did not have inhibitory zones after 48 h of cultivation. These results confirmed that methyl alcohol and metabolites of strain D-2 showed no antagonism against pathogen Pcc.

### Substrate Range and Degradation Capacity of Strain D-2

**FIGURE 2:**
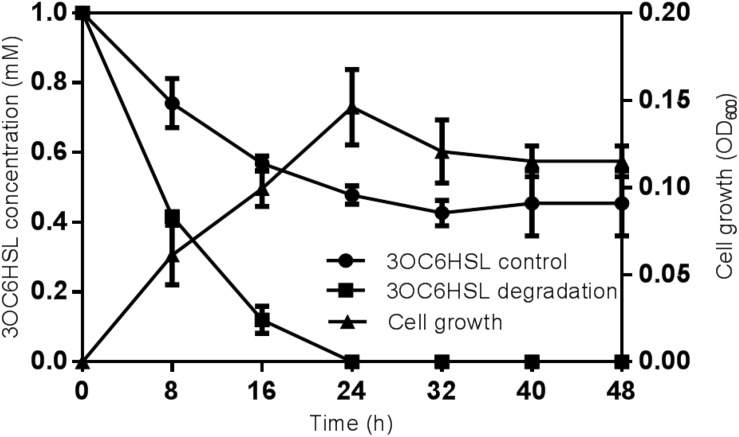
Degradation of 3OC6HSL during the growth of strain D-2. Black square, 3OC6HSL degradation; Black triangle, growth of strain D-2; Black rotundity, 3OC6HSL control.

The substrate range was investigated through biosensor strain *A. tumefaciens* NT1, which tested the ability of isolates to inactivate 3OC6HSL. Strain *O. intermedium* D-2 has the ability to utilize various AHLs as a substrate. Strain D-2 effectively inactivated AHLs including 3OC6HSL, 3OC8HSL, and 3OC12HSL (Supplementary Figure S5). The growth of strain D-2 in MSM with 3OC6HSL (1 mmol⋅L^–1^) as the sole carbon resource and its characteristics are shown in Figure 2. There was no lag phase in the accumulation of strain D-2, indicating that it utilized 3OC6HSL as a growth substance. Cell growth hiked from 0 to 24 h, and 3OC6HSL was completely degraded, with a concomitant increase of cell number during this period. Finally, strain D-2 grew slowly after 24 h, and the residual amount of 3OC6HSL was not detectable by HPLC at 24 h post-incubation. In contrast, the degradation of control was significantly lower (approximately 40%) after 48 h.

### Effect of the QQ Strain D-2 Against Pcc

**FIGURE 3:**
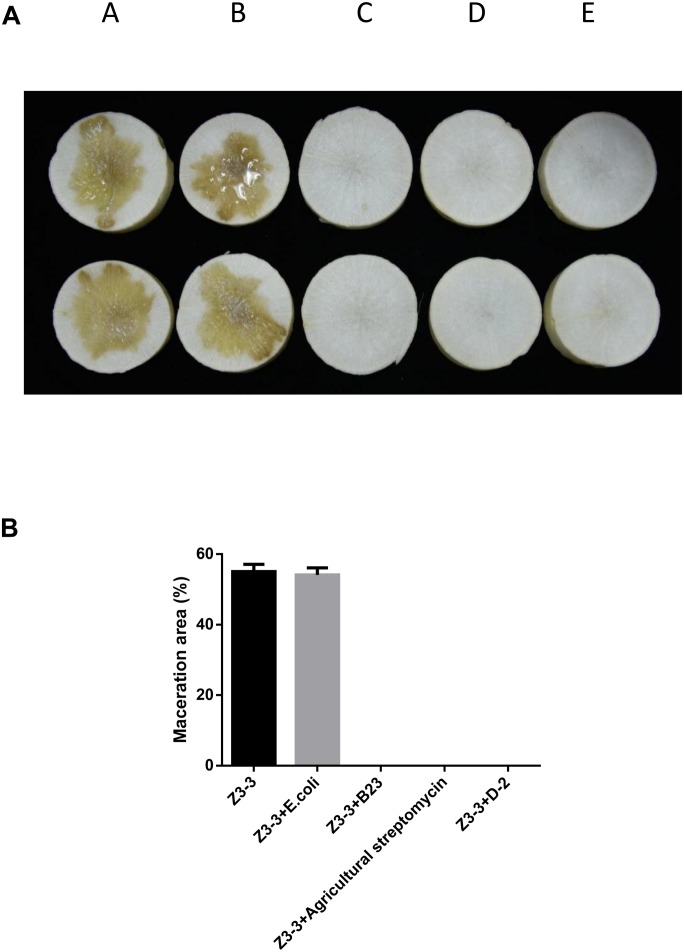
Maceration attenuating test of strain D-2 against soft rot disease on radish slices. *Bacillus thuringiensis* subsp. *israelensis* B23 and *Escherichia coli* DH5*α* served as positive and negative controls. **(A)** Panel A, Z3-3 alone on radish slices; Panel B, Z3-3 + *E. coli* DH5*α*; Panel C, Z3-3 + B23; Panel D, Z3-3 + agricultural streptomycin; Panel E, Z3-3 + D-2. **(B)** Numbers of maceration area in each treatment.

**FIGURE 4:**
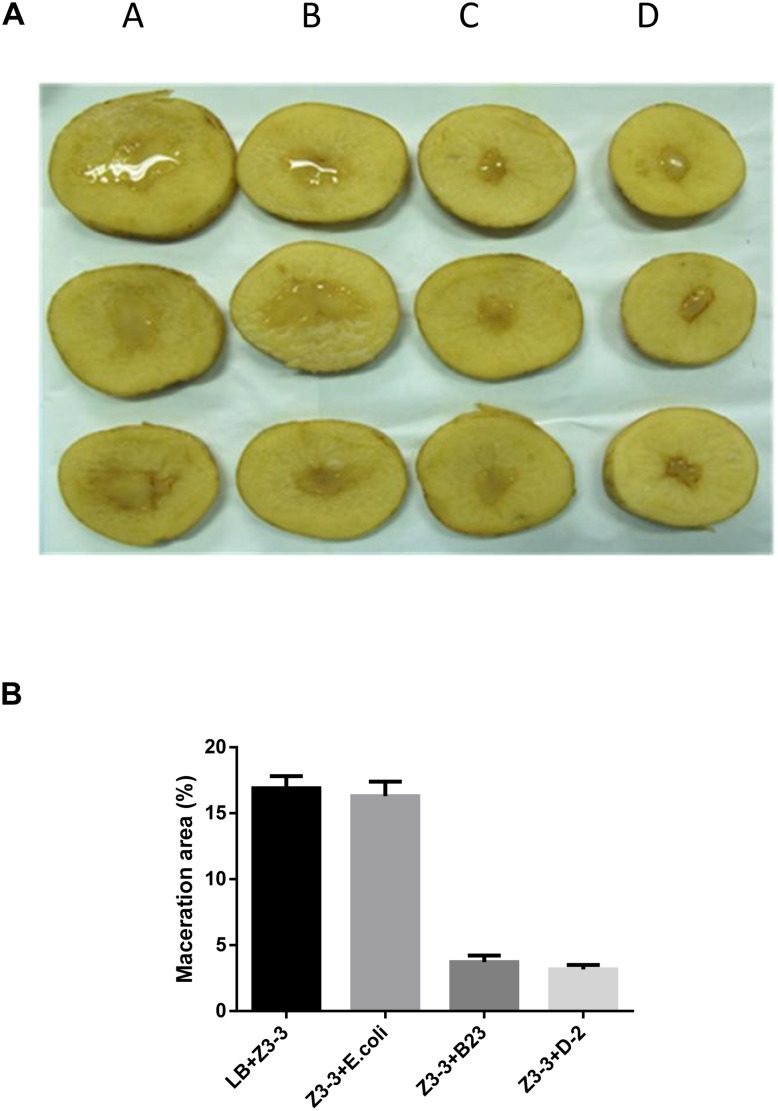
Maceration attenuating test of strain D-2 against soft rot disease on potato slices. *Bacillus thuringiensis* subsp. *israelensis* B23 and *Escherichia coli* DH5*α* served as positive and negative controls. **(A)** Panel A, Z3-3 alone on potato slices; Panel B, Z3-3 + *E. coli* DH5*α*; Panel C, Z3-3 + B23; Panel D, Z3-3 + D-2. **(B)** Numbers of maceration area in each treatment.

The maceration attenuating assay was conducted to evaluate the potential of QQ strain D-2 against Pcc. QQ strain, *E.coli* DH5*α*, B23, and agricultural streptomycin were separately mixed with Pcc Z3-3, and 2 μL of each mixture was injected into tuber slices of potato and radish. Results showed that strain D-2 exhibited 100% attenuation efficiency against Pcc on radish slices (Figure 3). Individual treatment of QQ strain D-2 significantly reduced disease severity caused by Pcc. Radish slices solely treated with Z3-3 or Z3-3 and *E. coli* DH5*α* resulted in severe disease incidence (Figure 3A, Panels A and B). Soft rot was not observed in radish slices treated with a mixture of Z3-3 and D-2 (Figure 3A, Panel E), similar to the treatment of Z3-3 and agricultural streptomycin (Figure 3A, Panel D) and to the treatment of Z3-3 and B23 (Figure 3A, Panel C). The diameter of the macerated region was consistent with the above results. The macerated region accounted for about 60% in Panels A and B, whereas, in Panels C, D, and E, no macerated region was observed (Figure 3B). Similar results were observed with the potato slices (Figure 4). QQ strain D-2 displayed promising efficiency of attenuating maceration produced by Pcc on the potato. Plant slices solely treated with Z3-3 or *E. coli* DH5*α* and Z3-3 led to severe disease incidence (Figure 4A, Panels A and B). Individual treatment of QQ strain D-2 substantially attenuated maceration on the potato. A slight decay (<3%) was observed in plant slices after treatment with a mixture of D-2 and Z3-3 (Figure 4A, Panel C), similar to the treatment of B23 and Z3-3 (Figure 4A, Panel D). In contrast, areas of macerated tissue (Figure 4B) were 17.9% and 17.5% in the treatment with Z3-3, and the mixture of *E. coli* DH5*α* and Z3-3, respectively. These results indicated that QQ strain D-2 is a potent biological control agent against pathogen Pcc and may have the potential to be used to control AHL-dependent bacterial pathogens.

### Biodegradation Products of Strain D-2

**FIGURE 5:**
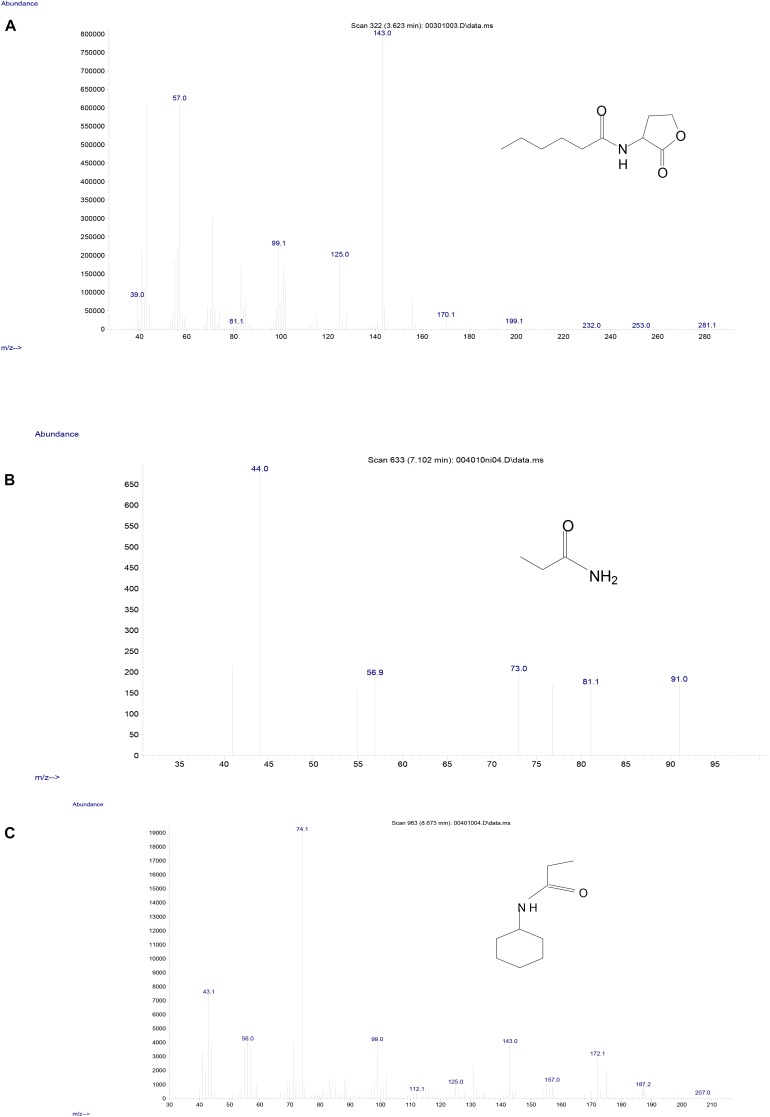
Mass spectra of AHL degradation products by strain D-2. **(A)** AHL; **(B)** Propanamide; **(C)** *N*-cyclohexyl-propanamide.

To investigate the degradation pathway of AHLs by strain D-2, cells of strain D-2 were cultivated in MSM (pH 6.5) containing 1 mmol⋅L^–1^ AHL. Samples were collected at 0, 8, 16, and 32 h. AHL and its degradation products were extracted, dried, resuspended in methanol, and analyzed through GC-MS to identify the metabolic products of the degradation process by *O. intermedium* D-2. Several GC peaks were detected as shown in Supplementary Figure S6. C6HSL and two main products were determined after background correction mass spectra and comparison with the control. Two main products were identified on the basis of the similarity of their fragment retention time (RT) and molecular ions to corresponding authentic compounds in the NIST library database. A significant compound (Supplementary Figure S6) was detected in all samples, which eluted at 3.623 min, displayed a characteristic mass fragment [M+] at m/z 143 (Figure 5A), and was identified as C6HSL. AHL disappeared concomitantly with the formation of two new compounds. One of the two compounds at RT 7.102 min showed a prominent protonated molecular ion at m/z 44 (Figure 5B) and was confirmed as propanamide (Supplementary Figure S7A). Another compound at RT 8.673 min showed a prominent protonated molecular ion at m/z 74 (Figure 5C) and was characterized as *N*-cyclohexyl-propanamide (Supplementary Figure S7B). It is noteworthy that the two metabolites were transient and faded away without any non-cleavable metabolites at the end of the experiment.

**FIGURE 6:**
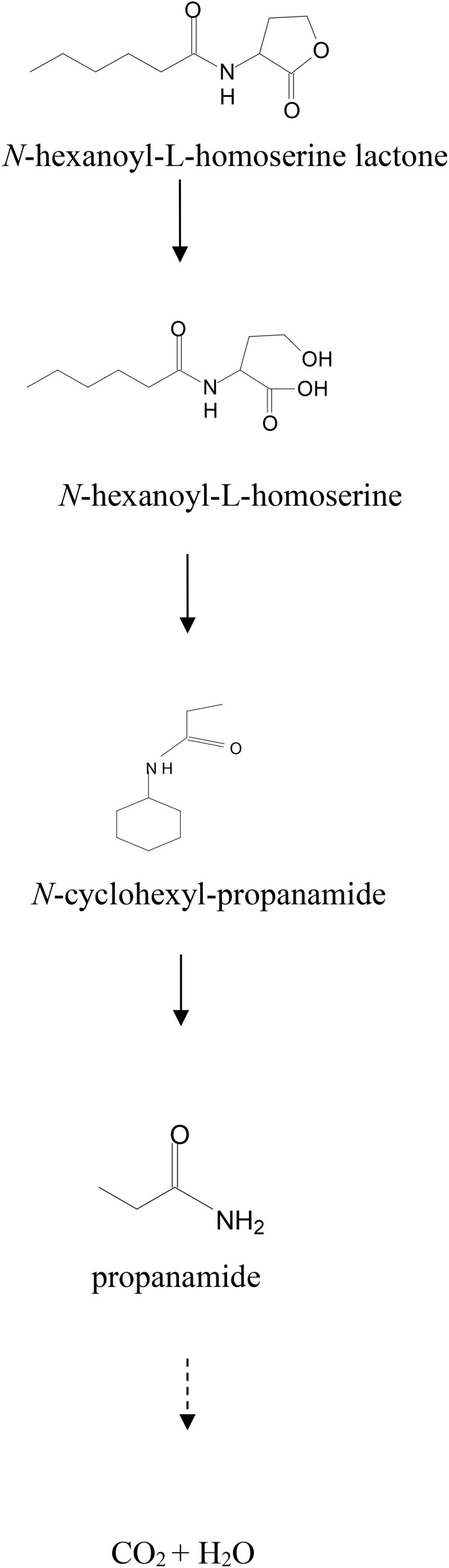
Proposed AHL degradation pathway in strain D-2.

The re-lactonization assay (Supplementary Figure S8A) indicated that strain D-2 inactivated AHL via lactonase cleaving the homoserine lactone ring of AHL molecules. Consequently, a novel degradation pathway of AHL in strain D-2 was proposed (Figure 6). As shown in Figure 6, AHL was first hydrolyzed by cleavage of the ester ring to produce *N*-hexanoyl-L-homoserine that was further enzymatically digested into *N*-cyclohexyl-propanamide and propanamide. Ultimately, C6HSL was degraded to carbon dioxide and water without any persistent accumulative product.

### Genome Characteristics

**FIGURE 7:**
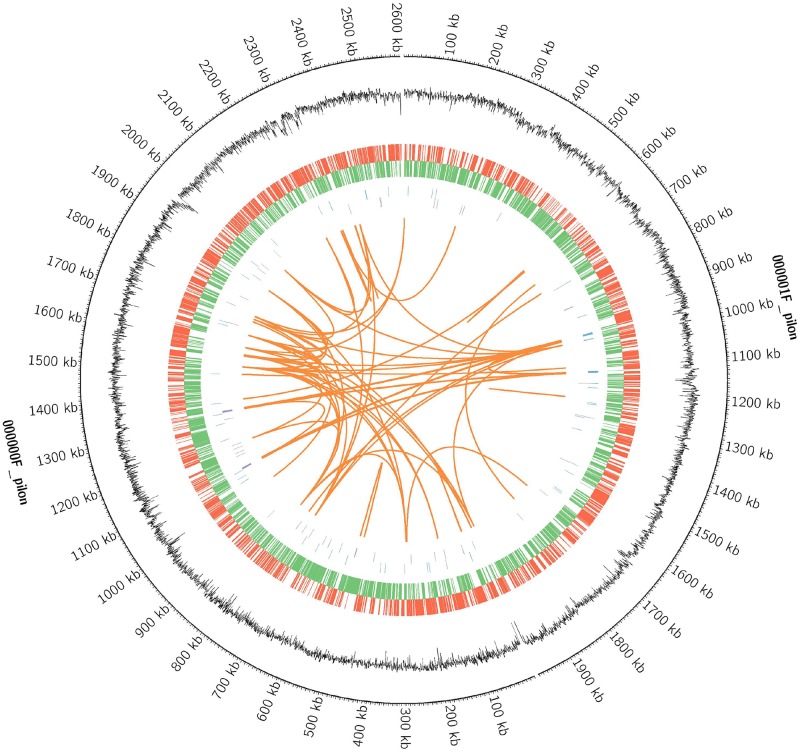
The circular genome diagram of *Ochrobactrum intermedium* D-2. Genomic analysis revealing the circular replicons in the genome of strain D-2. From the outside to the inside of the chromosome, the circles are as follows: circle 1, genomic location; circle 2, GC content; circle 3 and 4, location of encoding genes on the positive (red) and negative (green) strand; circles 5 and 6, locations of ncRNA on the positive (blue) and negative (purple) strand; circles 7, genomic long fragment repeats.

Whole-genome sequencing of strain D-2 was performed, and results are displayed in Figure 7 and Table 2. The genome of strain D-2 has a multireplicon structure, with a single circular chromosome. Locations of the coding sequence, ncRNA, and GC content are shown in Figure 7, and other detailed results are given in Table 2. The circular chromosome is 4, 594, 443 bp in size, with a GC content of 57.71%. It contains 4408 protein-coding sequences, 36 rRNA operons, and 64 tRNA genes. As shown in Table 2, these coding genes (CDSs) were identified and functionally characterized using the NR, CAZy, GO, KEGG, and COG databases, and 4383 CDSs, 452 CDSs, 554 CDSs, 2257 CDSs, and 3853 CDSs were functionally assigned to these five databases, respectively. Genome functional annotation of the strain D-2 against the COG database (Supplementary Figure S9) revealed 21 function classes, including the most abundant five: “amino acid transport and metabolism” (538 CDSs), “general function prediction only” (523 CDSs), “function unknown” (391 CDSs), “carbohydrate transport and metabolism” (339 CDSs), and “transcription” (332 CDSs). Classification of the biological processes, molecular function, and cellular component of this strain was performed by genome functional annotation against the GO database (Supplementary Figure S10). Genome functional annotation of strain D-2 against the KEGG database displayed six function types, including “cellular processes” (250 CDSs), “environmental information processing” (391 CDSs), “genetic information processing” (214 CDSs), “human diseases” (152 CDSs), “metabolism” (1841 CDSs), and “organismal systems” (53 CDSs) (Supplementary Figure S11). Classification of polysaccharide lyases, glycosyl transferases, glycoside hydrolases, auxiliary activities, carbohydrate esterases, and carbohydrate-binding modules of strain D-2 was performed by genome functional annotation against the CAZy database (Supplementary Figure S12).

**TABLE 2:** Major genome characteristics of the *Ochrobactrum intermedium* D-2.

Features	Number
Genome size (bp)	4,594,443
GC content (%)	57.71
Protein-coding genes (CDSs)	4408
rRNA	36
tRNA	64
Other RNA	45
Coding gene assigned to NR	4383
Coding gene assigned to GO	554
Coding gene assigned to KEGG	2257
Coding gene assigned to COGs	3853
Coding gene assigned to CAZy	452

### Cloning and Identification of a QQ Enzyme Gene in Strain D-2

The homology comparison between AidH enzyme, known QQ enzymes from *Ochrobactrum* sp. T63, and putative proteins of strain D-2 was carried out to determine the QQ enzyme of strain D-2. It was shown that the AidF protein displayed an 86.67% amino acid identity homologous with AidH (ACZ73823.1). Sequences of the gene *aidf* were PCR-amplified using primers mentioned above and fused in frame to a His_6_ tag in pET-28b. The recombinant strain *E. coli* BL21 (DE3) (pET-28b-*aidf*) possesses a degradation capacity toward 3OC6HSL. That is, *aidf* encodes a QQ enzyme named AidF.

The recombinant strain *E. coli* BL21 (DE3) (pET-28b-*aidf*) was obtained to express AidF protein. The purified His_6_-AidF protein had an apparent molecular mass of approximately 30 kDa upon 20% sodium dodecyl sulfate-polyacrylamide gel electrophoresis (SDS-PAGE), and this is consistent with its predicted molecular weight (29.3 kDa). Furthermore, the purified His_6_-AidF displayed a degradation capacity toward 3OC6HSL.

### Characterization of AHL Lactonase

To determine whether strain D-2 inactivated 3OC6HSL via lactonase and whether AidF protein is a type of lactonase, a mixture of the QQ strain D-2 culture and 3OC6HSL and a mixture of AidF protein and 3OC6HSL were incubated and acidified (pH = 2) to reconstruct the lactonase cleavage site. Results showed that the 3OC6HSL was inactivated by strain D-2 and the AidF protein (Supplementary Figure S8A, Panel D, and Supplementary Figure S8B, Panel D) and reconstructed after acidification (Supplementary Figure S8A, Panel H, and Supplementary Figure S8B, Panel H). The lactonase enzyme restored the structure of the lactone ring under acidic conditions. It is similar to lactonase (AiiA) containing *B. thuringiensis* subsp. *israelensis* B23, which can inactivate 3OC6HSL. The results of this study confirmed that the degradation activity of strain D-2 was due to the presence of a lactonase. Furthermore, the results of the acidification assay evidenced that this protein showed the character of AHL lactonase (Supplementary Figure S8B).

## Discussion

QS is a cell-to-cell communication (bacterial talk) system that coordinates the behavior of the bacteria within population through chemical signals (Bassler, 1999). QS is widely present in microorganisms and modulates the expression of specific genes, especially related to the virulence of pathogens (biofilm formation). Therefore, these QS systems are potential targets for anti-virulence therapies. The QS-based QQ is a new therapeutic strategy against plant diseases (Koh et al., 2013). QQ can be achieved by inhibiting the synthesis or detection of signal molecules, enzyme-catalyzed degradation, or the modification of signal molecules (Czajkowski and Jafra, 2009). Degradation of signal molecules by QQ bacteria is one of the most effective and non-toxic methods of QQ. QQ bacteria or the QQ enzyme-based interruption of QS systems is a novel strategy that shuts down the expression of pathogenicity genes without killing the pathogen or limiting its growth (Fetzner, 2015; Grandclement et al., 2016). This technique can replace traditional antibiotic treatments. QQ bacteria and QQ enzymes of AHL have been thoroughly investigated by various researchers (Du et al., 2014; Garge and Nerurkar, 2016). However, most QQ bacteria of AHL are not suitable for practical application due to their low efficiency and environmental adaptation.

In this study, a new soil bacterial isolate was screened and selected on the basis of degradation potential and identified as *O. intermedium* D-2 (strain D-2). The present work investigated the AHL degrading ability of *O. intermedium*. The genome sequence of strain D-2 was 4, 594, 443 bp with 57.71% GC content, and approximately 4408 coding genes (CDSs) were identified. Furthermore, an QQ enzyme, AidF protein, was identified from *O. intermedium.* AHL lactonases or acylases are the main QQ enzymes, which hydrolyze the AHL lactone ring (Zhang et al., 2019). Hydrolysis of AHL yielded *N*-acylhomoserine that can be restored at acidic pH to *N*-acyl homoserine lactone (Yates et al., 2002). According to this phenomenon, it has been demonstrated that AidF protein functions as AHL lactonases. This study also revealed that the *O. intermedium* D-2-based degradation product of AHL can be restored at acidic pH. Results suggest that intracellular enzymes of *O. intermedium* can degrade AHL. The majority of the AHL-degrading enzymes are either cytoplasmic or intracellular and utilize a wide variety of substrates (Kim et al., 2005; Liu et al., 2007; Czajkowski and Jafra, 2009; Zhang et al., 2019). AHL and their degradation products have acted as sources of carbon and nitrogen. Similar results were also observed in strain D-2. Some microbes can utilize fatty acids released from AHL as a source of energy (Park et al., 2003). Many QS signal molecule-degrading microbes have been found in nature, including *Gordonia* sp. (Soler et al., 2018), *Bacillus* sp. (Dong et al., 2002; Lee et al., 2002; Ulrich, 2004; Soler et al., 2018; Bhatt et al., 2020), *Streptomyces* sp. (Park et al., 2005; Devaraj et al., 2017), *Micromonospora* sp. (Devaraj et al., 2017), *Agrobacterium* sp. (Carlier et al., 2003) and *Rhodococcus* sp. (Uroz et al., 2005; Park et al., 2006; Barbey et al., 2013; Mueller et al., 2015). QQ enzymes produced by these QQ bacteria strains include lactonase, acylase, oxidoreductase, and paraoxonase. Lactonase has been found in a range of bacterial species (Dong et al., 2002; Bhatt et al., 2019; Zhang et al., 2019). However, little information is available about QQ bacteria in *Ochrobactrum* sp. and their potential role in attenuating maceration produced by Pcc, which is modulated by the QS system through the 3OC6HSL signal molecule. The potential of *O. intermedium* strain D-2 as QQ bacteria was explored for the first time in this study, and it was found effective similar to earlier reported genera.

Naturally, diverse organisms produce AHL-degrading enzymes, but only a few bacterial isolates have been reported as agents attenuating maceration of agricultural pathogenesis (Jafra et al., 2006; Cirou et al., 2007). Therefore, the main focus of the study was to explore *O. intermedium* D-2 as an agent to attenuating maceration of Pcc pathogenesis. The QS signaling molecule of Pcc is 3OC6HSL, and the *O. intermedium* D-2 exhibited the potential of degrading various AHL during in vitro assay. The result of the experiment established that *O. intermedium* D-2 utilized QS signals of Pcc, which mainly play a role in the reduction of virulence through biofilm formation (Molina et al., 2003; Gantner et al., 2006). It was confirmed that this phenomenon was not due to antagonism against Pcc. *O. intermedium* D-2 markedly attenuated bacterial virulence on host plants by reducing the accumulation of endogenous AHL. This phenomenon is different from the antagonistic bacteria. In addition, QQ strain D-2 demonstrated successful preventive and curative effects on the attenuating maceration of Pcc on host plants. The broad substrate range of QQ strain D-2 will facilitate further investigation of their potential in degrading QS signals. In the future, strain D-2 will be helpful in reducing the pathogenesis of soft rot pathogen and other AHL-dependent bacteria. The present study highlights the utility of QQ strain D-2 as an agent for AHL degradation and the control of AHL-dependent bacterial pathogens.

## Conclusion

In this work, we identified a QQ candidate, *O. intermedium* D-2, against soft rot pathogen Pcc. QQ strain D-2 showed superior AHL degradation activity and effectively degraded a wide range of AHLs, including C6HSL, 3OC6HSL, 3OC8HSL, and 3OC12HSL. Strain D-2 harbors the metabolic pathway for the complete degradation and metabolism of AHL. Moreover, the application of strain D-2 can substantially reduce the disease severity of Pcc on host plants. Furthermore, a QQ enzyme, AidF protein, was identified from strain D-2 through whole-genome analysis. It has been demonstrated that the protein AidF functions as an AHL-lactonase in the re-lactonization assay. These findings unveil the biochemical basis of a highly efficient AHL-degrading bacterial isolate and present the potential to attenuate Pcc virulence through quorum quenching. In the future, further studies will be performed to investigate the relationship between the *aidf* gene and virulence factors of Pcc.

## Data Availability Statement

The raw data supporting the conclusions of this article will be made available by the authors, without undue reservation, to any qualified researcher.

## Author Contributions

SC and LZ designed the experiment. XF, TY, and QL performed the experiment. XF and TY analyzed data. XF, TY, PB, and SC wrote the manuscript.

## Conflict of Interest

The authors declare that the research was conducted in the absence of any commercial or financial relationships that could be construed as a potential conflict of interest.
