# Supplementary material for: Potential of a Quorum Quenching Bacteria Isolate Ochrobactrum intermedium D-2 Against Soft Rot Pathogen Pectobacterium carotovorum subsp. carotovorum
Source: Front Microbiol. 2020 May 8;11:898. doi: 10.3389/fmicb.2020.00898 (PMC7227377; doi:10.3389/fmicb.2020.00898)
Supplement: Supplementary file 1 [file Data_Sheet_1.doc]

**Potential of a quorum quenching bacteria isolate *Ochrobactrum intermedium* D-2 against soft rot pathogen *Pectobacterium carotovorum* subsp. *carotovorum***

Xinghui Fan^1,2^, Tian Ye^1,2^, Qiting Li^1,2^, Pankaj Bhatt^1,2^, Lianhui Zhang^1,2^, Shaohua Chen^1,2*^

^1^State Key Laboratory for Conservation and Utilization of Subtropical Agro-bioresources, Guangdong Province Key Laboratory of Microbial Signals and Disease Control, Integrative Microbiology Research Centre, South China Agricultural University, Guangzhou 510642, China;

^2^Guangdong Laboratory for Lingnan Modern Agriculture, Guangzhou 510642, China

**Running title:** Attenuation of Pcc virulence by *O. intermedium* D-2

^*^**Correspondence:**

Shaohua Chen

[shchen@scau.edu.cn](mailto:shchen@scau.edu.cn)

**Figure S1** Chemical structures of various *N*-acyl homoserine lactones (AHLs) used in this study. A, *N*-hexanoyl-L-homoserine lactone (C6HSL); B, *N*-(3-oxohexanoyl)-L-homoserine lactone (3OC6HSL); C,  *N*-(3-oxooctanoyl)-L-homoserine lactone (3OC8HSL); D, *N*-(3-oxododecanoyl)-L-homoserine lactone (3OC12HSL).


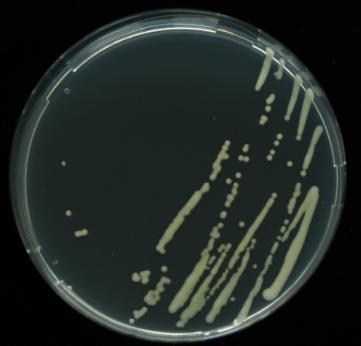


a


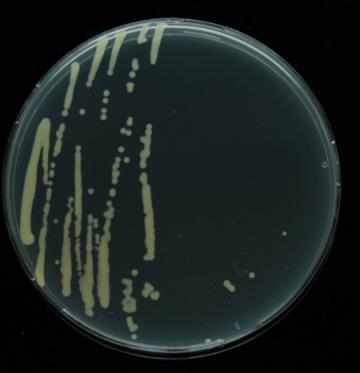


b


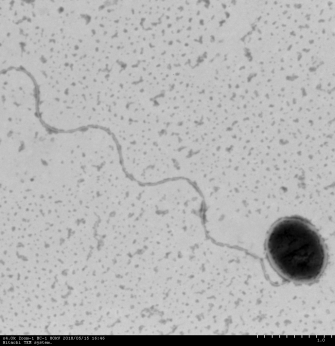


c


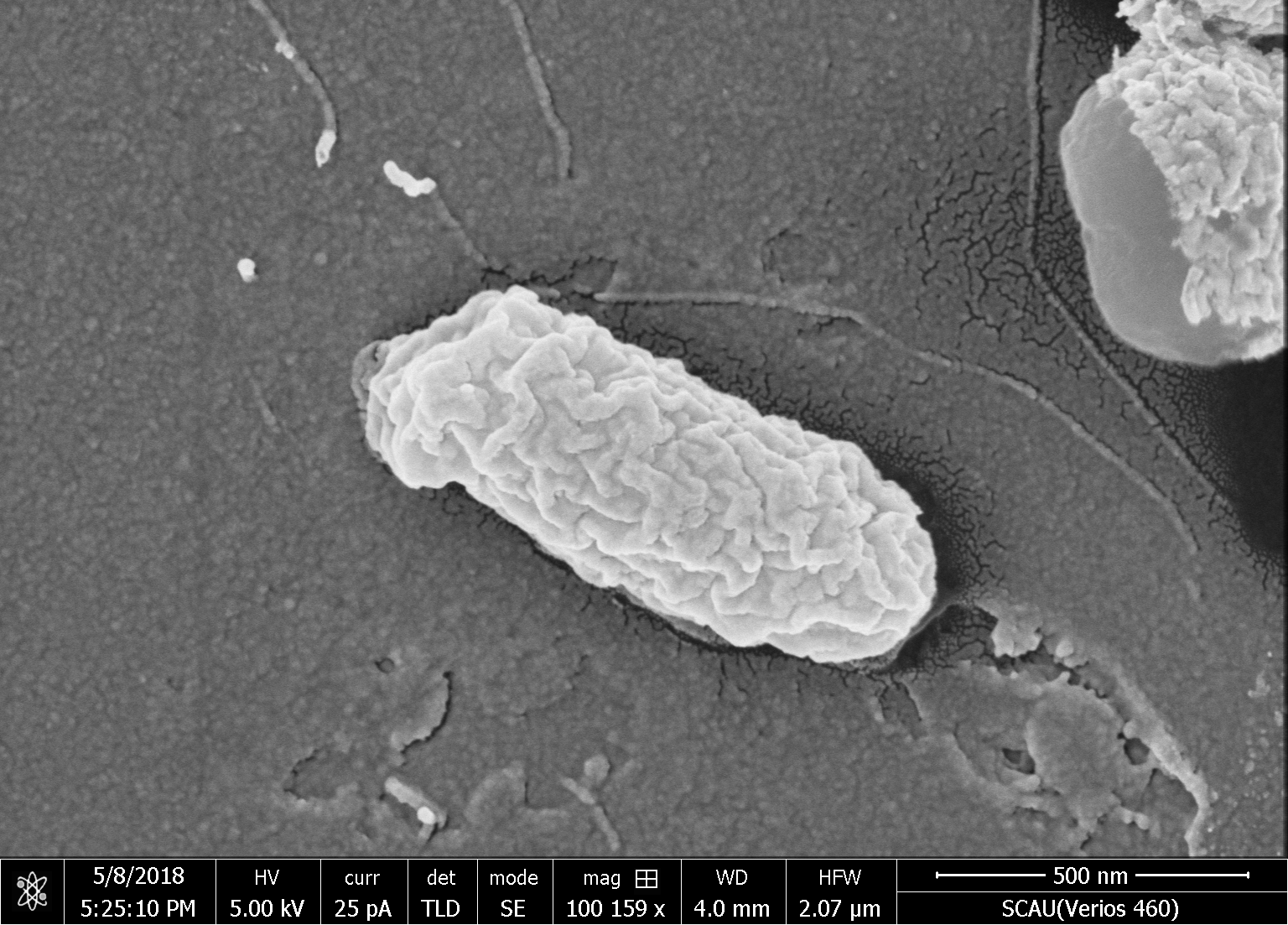


d

**Figure S2** Morphological characteristics of *Ochrobactrum intermedium* D-2. a: Colony morphology (front); b: Colony morphology (back); c: Morphological characteristics under scanning electron microscope (2000x); d: Morphological characteristics under scanning electron microscope (4000x).

**Figure S3** Antibiotic sensitivity of *Ochrobactrum intermedium* D-2.


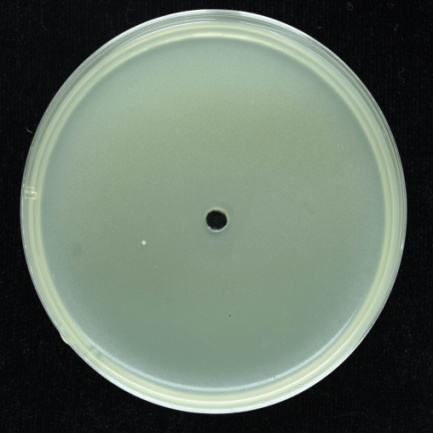

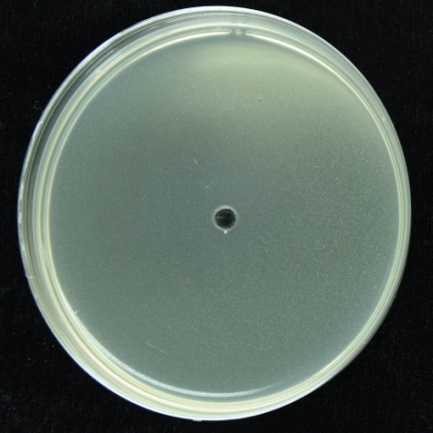


**a b**

**Figure S4** Antagonism test between *Pectobacterium carotovorum* subsp. *carotovorum* strain Z3-3 and strain D-2. a: Inoculation of 20 μL methyl alcohol; b: Inoculation of 20 μL metabolites of strain D-2.


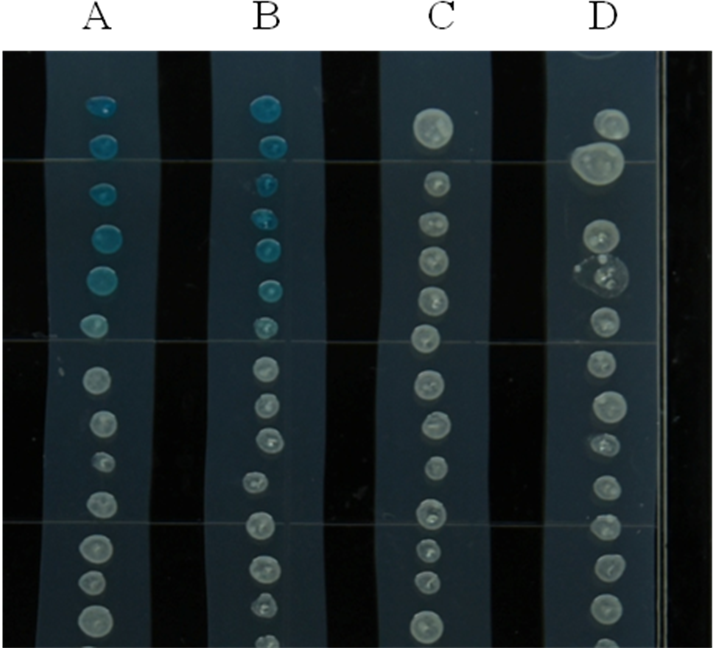


**A**: Blank control: only contiaining 3OC6HSL (20 μmol·L^-1^)

**B**: Negative control: degradation of 3OC6HSL (20 μmol·L^-1^) by *Escherichia coli* DH5*α*

**C**: Positive control: degradation of 3OC6HSL (20 μmol·L^-1^) by *Bacillus* *thuringiensis* subsp. *israelensis* B23

**D**: Degradation of 3OC6HSL (20 μmol·L^1^) by *Ochrobactrum intermedium* D-2

a

**
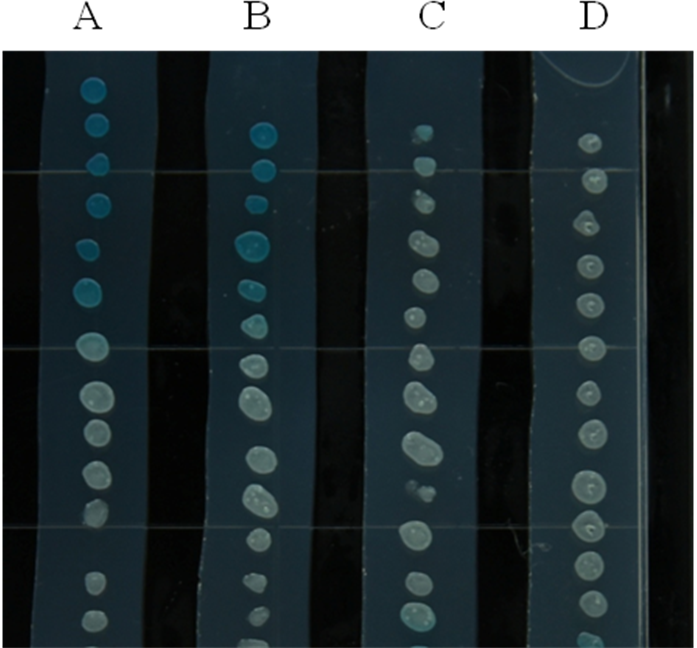
**

b

**A**: Blank control: only contiaining 3OC8HSL (20 μmol·L^-1^)

**B**: Negative control: degradation of 3OC8HSL (20 μmol·L^-1^) by *Escherichia coli* DH5*α*

**C**: Positive control: degradation of 3OC8HSL (20 μmol·L^-1^) by *Bacillus* *thuringiensis* subsp. *israelensis* B23

**D**: Degradation of 3OC8HSL (20 μmol·L^1^) by *Ochrobactrum intermedium* D-2

**
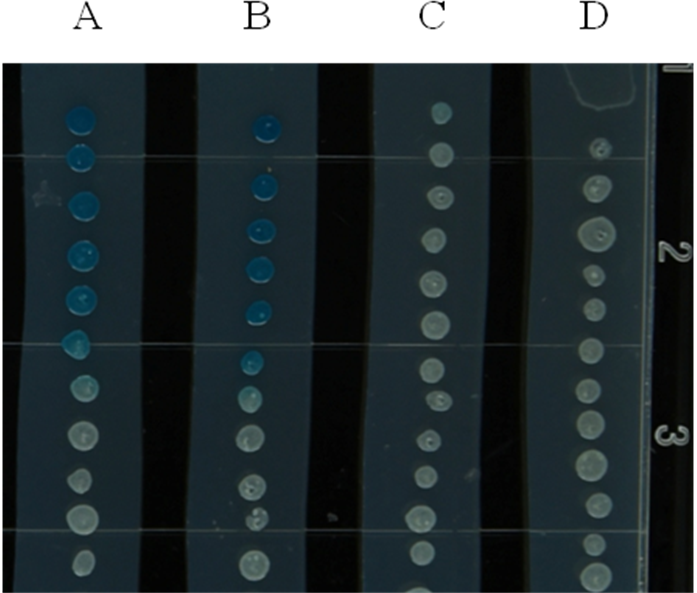
**

**A**: Blank control: only contiaining 3OC12HSL (20 μmol·L^-1^)

**B**: Negative control: degradation of 3OC12HSL (20 μmol·L^-1^) by *Escherichia coli* DH5*α*

**C**: Positive control: degradation of 3OC12HSL (20 μmol·L^-1^) by *Bacillus* *thuringiensis* subsp. *israelensis* B23

**D**: Degradation of 3OC12HSL (20 μmol·L^1^) by *Ochrobactrum intermedium* D-2

c

**Figure S5** Degradation of different AHLs by *Ochrobactrum intermedium* D-2. *Bacillus thuringiensis* subsp. *israelensis* B23 and *Escherichia coli* DH5*α* served as positive and negative controls. (a) Degradation of *N*-(3-oxohexanoyl)-L-homoserine lactone (3OC6HSL); (b) Degradation of *N*-(3-oxooctanoyl)-L-homoserine lactone (3OC8HSL); (c) Degradation of *N*-(3-oxododecanoyl)-L-homoserine lactone (3OC12HSL).

**Figure S6** Full scan mass spectrum of the degradation products by *Ochrobactrum intermedium* D-2.

**a**

**b**

**Figure S7** Mass spectra of degradation products of AHL by *Ochrobactrum intermedium* D-2 in the NIST library database. a: Propanamide; b: *N*-cyclohexyl-propanamide.


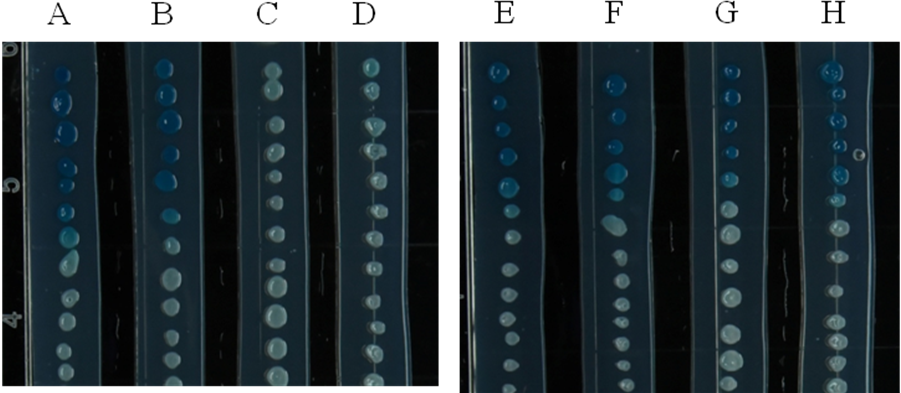


**A**: Blank control: only containing 3OC6HSL; **B**: Negative control: degradation of 3OC6HSL by *Escherichia coli* DH5*α*; **C**: Positive control: degradation of 3OC6HSL by *Bacillus* *thuringiensis* subsp. *israelensis* B23; **D**: Degradation of 3OC6HSL by *Ochrobactrum intermedium* D-2. E, F ,G, and H are acidifying A, B, C, and D, respectively.

a


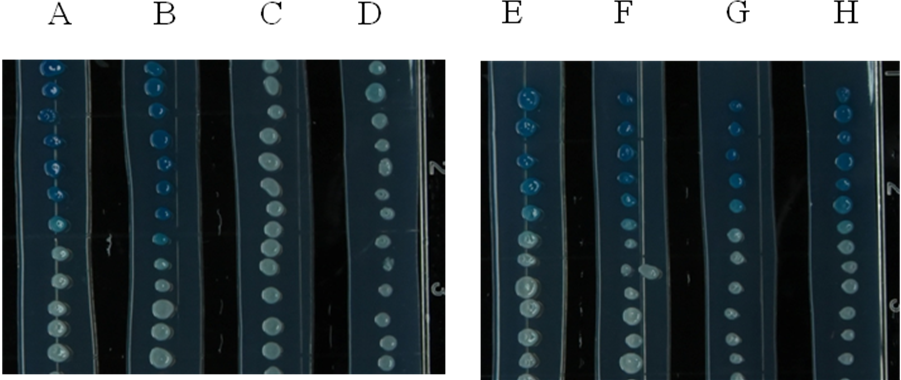


**A**: Blank control: only containing 3OC6HSL; **B**: Negative control: degradation of 3OC6HSL by crude enzyme of *Escherichia coli* DH5*α*; **C**: Positive control: degradation of 3OC6HSL by crude enzyme of *Bacillus* *thuringiensis* subsp. *israelensis* B23; **D**: Degradation of 3OC6HSL by the protein AidF. E, F ,G, and H are acidifying A, B, C, and D, respectively.

b

**Figure S8** Re-lactonisation assay of *N*-(3-oxohexanoyl)-L-homoserine lactone (3OC6HSL). (a) Degradation of 3OC6HSL by *Ochrobactrum intermedium* D-2. *Bacillus thuringiensis* subsp. *israelensis* B23 and *Escherichia coli* DH5*α*. (b) Degradation of 3OC6HSL by crude enzyme of *O. intermedium* D-2. *B. thuringiensis* subsp. *israelensis* B23 and *E. coli* DH5*α.*


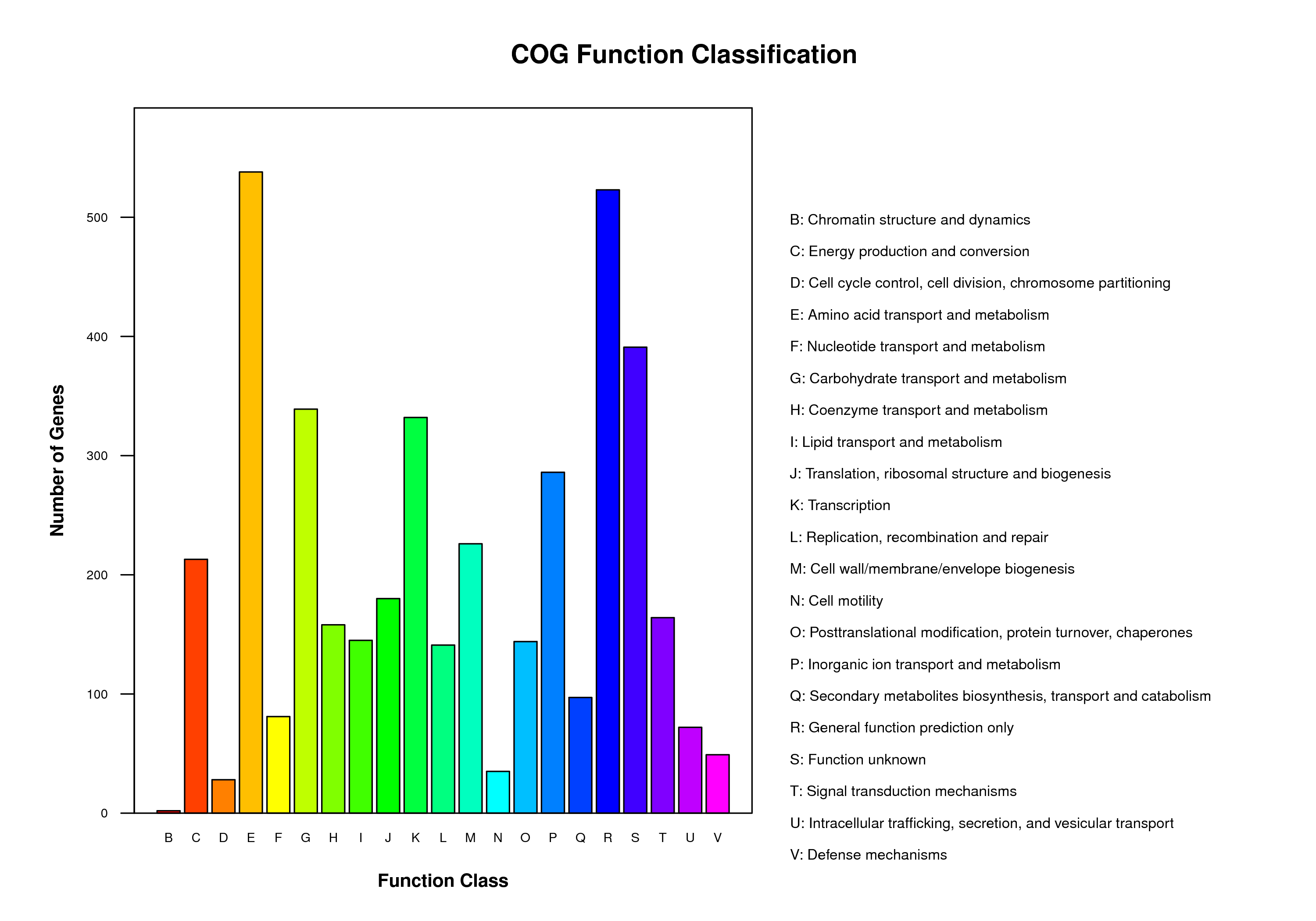


**Figure S9** Functional annotations of the *Ochrobactrum intermedium* D-2 genome against the COG database.


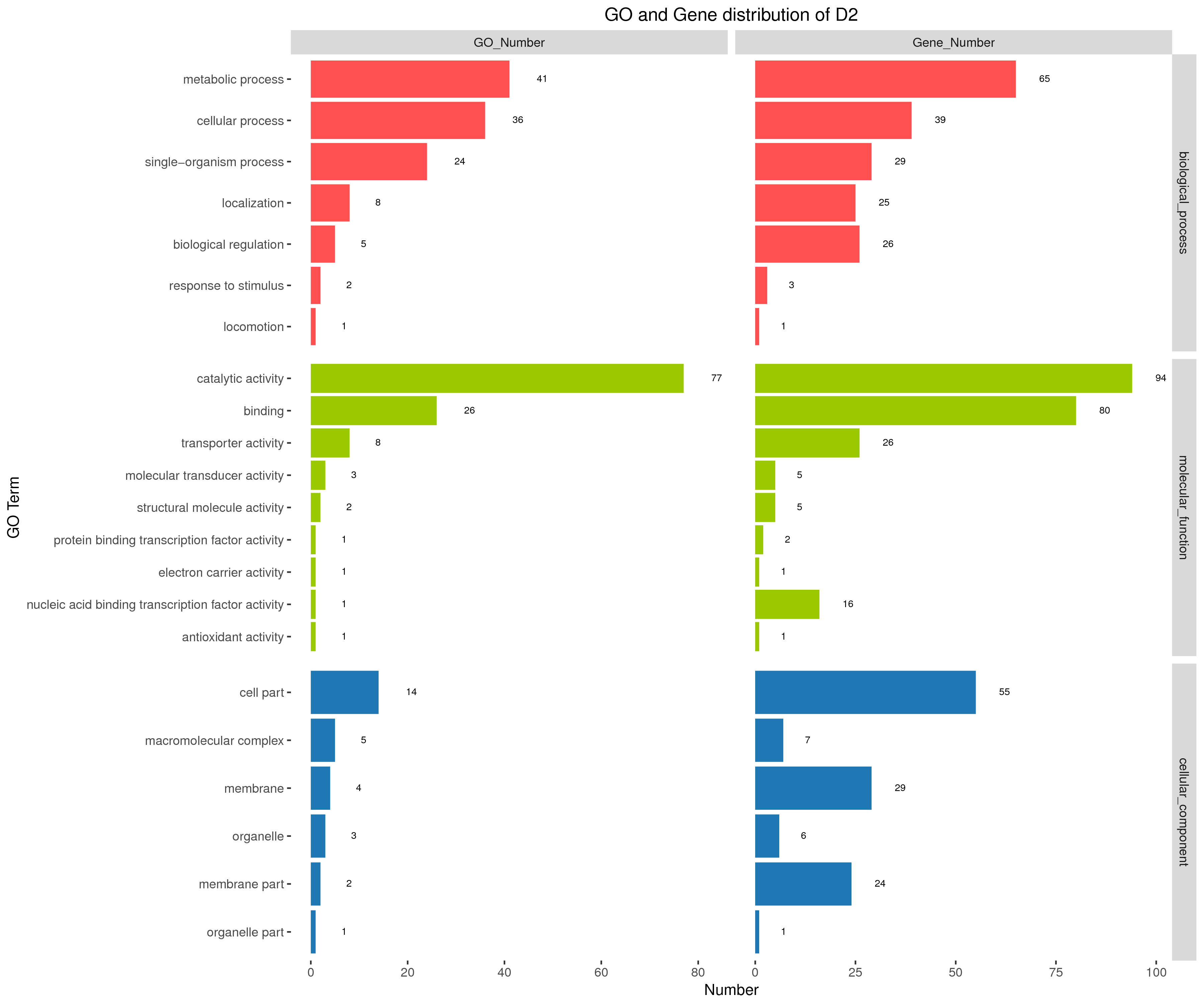


**Figure S10** Functional annotations of the *Ochrobactrum intermedium* D-2 genome against the GO database.


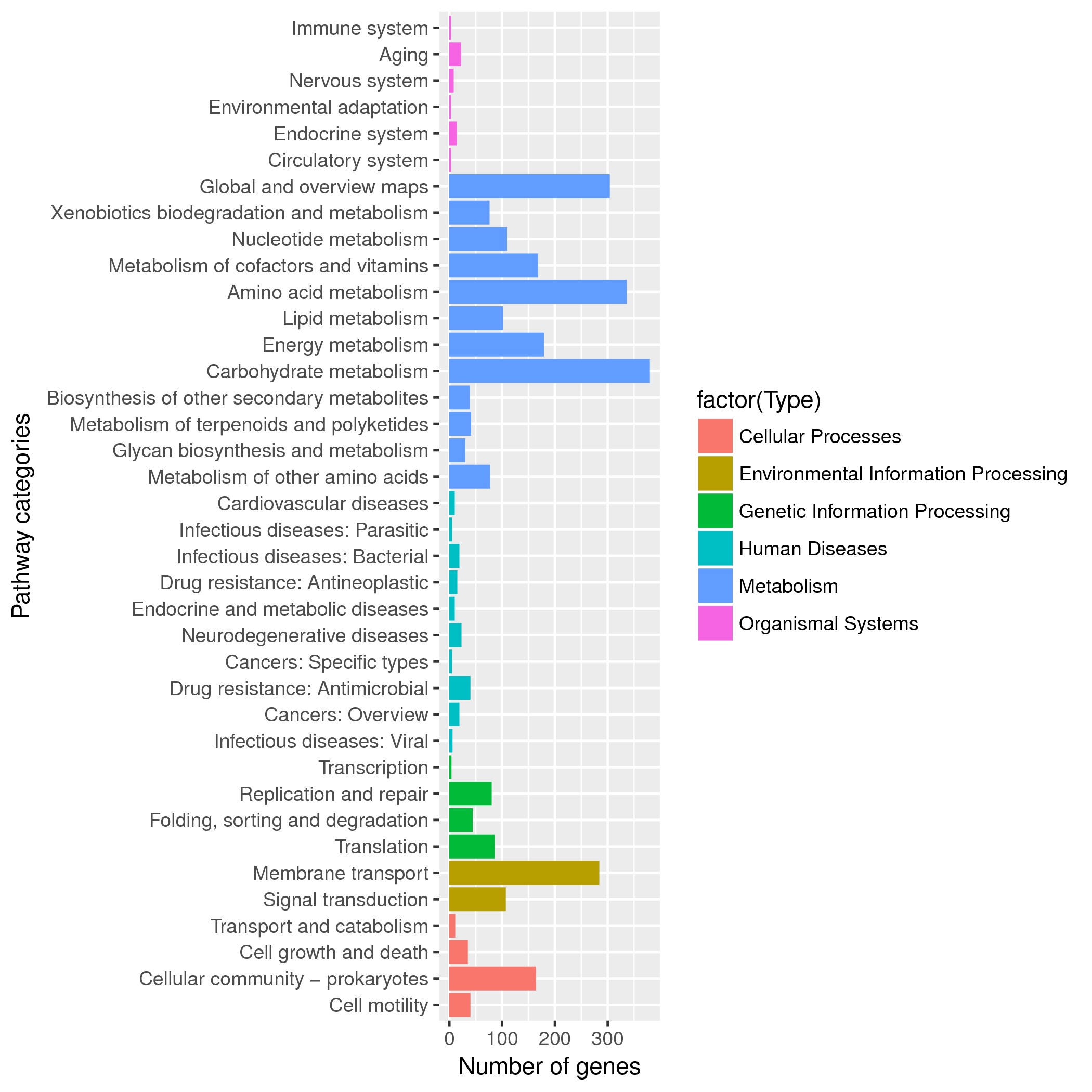


**Figure S11** Functional annotations of the *Ochrobactrum intermedium* D-2 genome against the KEGG database.


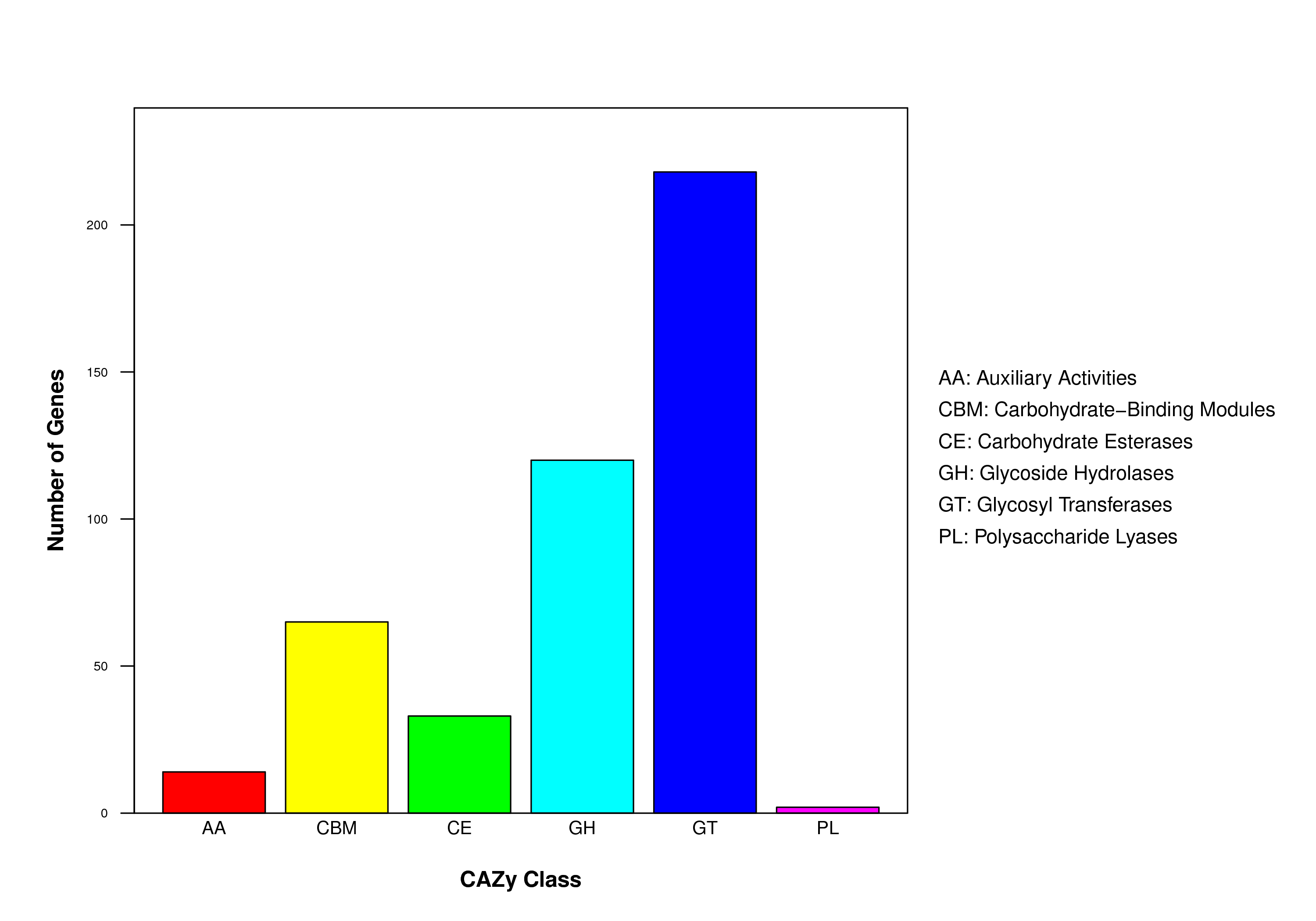


**Figure S12** Functional annotations of the *Ochrobactrum intermedium* D-2 genome against the CAZy database.

**Table S1** Isolation and screening of AHL-degrading bacterial strains.

| Strains | AHL degradation (%) |
| --- | --- |
| D-1 | 85.8 |
| D-2 | 100 |
| D-3 | 63.6 |
| D-4 | 54.3 |
| D-5 | 33.5 |
| D-6 | 74.1 |
| D-7 | 81.0 |
| D-8 | 66.7 |

**Table S2** Physio-biochemical characteristics of *Ochrobactrum intermedium* D-2.

| Characteristics | Results | Characteristics | Results |
| --- | --- | --- | --- |
| Gram staining | – | Anaerobic test | – |
| Gelatin liquefaction | – | Oxidase | ＋ |
| Catalase | ＋ | Dextrin | – |
| Sodium butyrate | ＋ | D-Maltose | ＋ |
| D-Trehalose | ＋ | D-Cellobiose | ＋ |
| Gentiobiose | ＋ | Sucrose | ＋ |
| D-Turanose | ＋ | Stachyose | – |
| D-Raffinose | – | *α*-D-Lactose | – |
| D-Melibiose | – | *β*-Methyl-D-Glucoside | – |
| N-Acetyl-*β*-D-Mannosamine | – | N-Acetyl-D-Glucosamine | ＋ |
| N-Acetyl Neuraminic Acid | – | *α*-D-Glucose | ＋ |
| D-Mannose | ＋ | D-Fructose | ＋ |
| D-Galactose | ＋ | 3-Methyl Glucose | – |
| D-Fucose | ＋ | L-Fucose | ＋ |
| L-Rhamnose | ＋ | D-Sorbitol | ＋ |
| D-Mannitol | – | Glycerol | – |
| D-Glucose-  6-PO4 | – | D-Fructose-6-PO4 | – |
| D-Aspartic Acid | – | Gelatin | – |
| L-Glutamic Acid | ＋ | L-Histidine | ＋ |
| L-Pyroglutamic  Acid | – | L-Serine | ＋ |
| D-Galacturonic  Acid | ＋ | L-Galactonic  Acid Lactone | ＋ |
| D-Glucuronic  Acid | ＋ | Mucic Acid | – |
| Quinic Acid | – | D-Saccharic Acid | – |
| p-Hydroxy-  Phenylacetic  Acid | – | D-Lactic Acid  Methyl Ester | – |
| Citric Acid | ＋ | *α*-Keto-Glutaric  Acid | ＋ |
| D-Malic Acid | ＋ | L-Malic Acid | ＋ |
| Tween 40 | – | Acetoacetic Acid | – |
| Acetic Acid | ＋ | Formic Acid | – |
| pH 5 | – | 1% NaCl | ＋ |
| 8% NaCl | – | 1% Sodium  Lactate | ＋ |
| D-Serine | – | Rifamycin SV | ＋ |
| Minocycline | – | Lincomycin | ＋ |
| Guanidine HCl | ＋ | Niaproof 4 | – |
| Vancomycin | ＋ | Tetrazolium  Violet | ＋ |
| Tetrazolium  Blue | ＋ | Potassium  Tellurite | ＋ |
| Aztreonam | ＋ |  |  |

Note: ＋, tested positive; －, tested negative.
